# A longitudinal study assessing differences in causes of death among housed and homeless people diagnosed with HIV in San Francisco

**DOI:** 10.1186/s12889-019-7817-7

**Published:** 2019-11-01

**Authors:** Nancy A. Hessol, Monica Eng, Annie Vu, Sharon Pipkin, Ling C. Hsu, Susan Scheer

**Affiliations:** 1 Department of Clinical Pharmacy, University of California, 3333 California Street, Suite 420, San Francisco, CA 94118 USA; 2 Department of Public Health, 25 Van Ness Avenue, Suite 500, San Francisco, CA 94102 USA

**Keywords:** Cause of death, HIV, Homeless, Housing, Mental health, Mortality

## Abstract

**Background:**

San Francisco has implemented several programs addressing the needs of two large vulnerable populations: people living with HIV and those who are homeless. Assessment of these programs on health outcomes is paramount for reducing preventable deaths.

**Methods:**

Individuals diagnosed with HIV/AIDS and reported to the San Francisco Department of Public Health HIV surveillance registry, ages 13 years or older, who resided in San Francisco at the time of diagnosis, and who died between January 1, 2002, and December 31, 2016 were included in this longitudinal study. The primary independent variable was housing status, dichotomized as ever homeless since diagnosed with HIV, and the dependent variables were disease-specific causes of death, as noted on the death certificate. The Cochran-Armitage test measured changes in the mortality rates over time and unadjusted and adjusted Poisson regression models measured prevalence ratios (PR) and 95% confidence intervals (CI) for causes of death.

**Results:**

A total of 4158 deceased individuals were included in the analyses: the majority were male (87%), ages 40–59 years old at the time of death (64%), non-Hispanic White (60%), men who have sex with men (54%), had an AIDS diagnosis prior to death (87%), and San Francisco residents at the time of death (63%). Compared to those who were housed, those who were homeless were more likely to be younger at time of death, African American, have a history of injecting drugs, female or transgender, and were living below the poverty level (all *p* values < 0.0001). Among decedents who were SF residents at the time of death, there were declines in the proportion of deaths due to AIDS-defining conditions (*p* < 0.05) and increases in accidents, cardiomyopathy, heart disease, ischemic disease, non-AIDS cancers, and drug overdoses (*p* < 0.05). After adjustment, deaths due to mental disorders (aPR = 1.63, 95% CI 1.24, 2.14) were more likely and deaths due to non-AIDS cancers (aPR = 0.63, 95% CI 0.44, 0.89) were less likely among those experiencing homelessness.

**Conclusions:**

Additional efforts are needed to improve mental health services to homeless people with HIV and prevent mental-health related mortality.

## Background

Use of effective antiretroviral therapy (ART) has enabled many people living with HIV (PLWH) to have healthier and longer lives. In San Francisco the three-year survival time among people with stage 3 HIV (AIDS) increased from 52% in the pre-ART years of 1990–1995 to 90% in the ART era years of 2006–2015 [1]. Adoption of ART has also led to a change in the causes of death among PLWH. This change is evident in San Francisco where the proportion of deaths among PLWH in which HIV was listed as a cause of death declined from 69.8% in the years 2006–2009 to 59.0% in 2014–2017 [1]. Conversely, deaths due to heart disease and non-AIDS cancer increased from 23.2 and 14.7%, respectively, to 29.9 and 20.3%, respectively, for these same time periods.

While cause-specific mortality among PLWH is well studied, there is little information on cause-specific mortality for homeless PLWH. In 2016 there were an estimated 291 homeless PLWH in San Francisco [1]. A city-wide point-in-time count in 2017 estimated that 4353 people, regardless of HIV status, were unsheltered in San Francisco and another 2505 individuals were marginally sheltered [2]. Separate studies of people living in Philadelphia, New York City, and Boston have reported that homeless individuals have higher mortality rates compared to the general population due to factors such as higher rates of psychiatric illness and substance abuse [3–5]. Carrying the additional burden of living with HIV puts people living with homelessness in a more vulnerable state.

Despite these comorbidities, the five-year HIV survival rate has improved over time for PLWH who are experiencing homelessness in San Francisco. Among all PLWH in San Francisco diagnosed with HIV from 2002 to 2006, the five-year survival rate for homeless individuals was 79% compared to 92% among housed individuals (*p* < 0.001) [6]. However, among persons diagnosed with HIV more recently from 2007 to 2011 there was no difference in the five-year survival rate between homeless and housed people (*p* = 0.40) [6]. A study analyzing a permanent supportive housing program in San Francisco found that among 6558 AIDS cases diagnosed from 1996 to 2006 in San Francisco, the five-year survival rate for persons who were homeless was 67% compared to 81% among those who were housed under permanent supportive housing (*p* < 0.0001) [7]. In contrast to the improvement in mortality data, a 2017 study in San Francisco reported poorer HIV viral suppression among homeless PLWH. The study observed lower odds for achieving viral suppression with individuals living in shelters (adjusted odds ratio [OR] = 0.26, 95% CI 0.12, 0.59) and those living outdoors (adjusted OR = 0.15, 95% CI 0.08, 0.29) [8]. Even though several efforts have been made to provide stable housing and resources in San Francisco, homelessness continues to be a barrier to managing an HIV diagnosis.

Three interventions aimed to support persons experiencing homelessness in San Francisco were established between 1998 and 2006: Direct Access to Housing (DAH), Care Not Cash, and Mental Health Services Act (MHSA). DAH, also known as permanent supportive housing, was established in 1998 by the San Francisco Department of Public Health (DPH) Housing and Urban Health Section. The program aims to house low-income San Francisco residents who are homeless and have special needs such as mental health issues, alcohol or substance abuse, or complex medical conditions such as AIDS [9]. Additionally, the Care Not Cash program was established by the San Francisco Human Services Agency in 2004 and shifted the utilization of funds from cash grants given to the homeless to expanding mental health and substance abuse services [10]. The budget for such services and treatment beds saw an increase from $666,000 in 2004 to $1.2 million in 2008 and also led to an additional 1321 affordable units for the homeless [10]. Furthermore, in 2006, the California Department of Mental Health implemented MHSA, which added a state personal income tax surcharge of 1% on taxpayers with annual taxable incomes of more than $1 million (approximately 25,000 to 30,000 taxpayers) [11]. These funds were transferred to the new Mental Health Services Fund and were used to expand the county medical services such as psychiatric, counseling, and hospitalization to any Californians who lacked coverage for such care, as well as permanent supportive housing for homeless individuals with serious mental disorders [12].

Along the same time period that these programs were established, HIV prevention and care leaders in San Francisco also implemented programs to improve HIV care. In May 2006, San Francisco General Hospital (SFGH; San Francisco’s only municipal hospital) eliminated the requirement for written consent for HIV testing and added the HIV antibody test to the routine lab order form [13]. In 2010, the San Francisco DPH implemented universal Test and Treat in all publicly funded clinics, recommending that all patients initially diagnosed with HIV be offered ART regardless of their CD4 T lymphocyte (CD4) count. Prior to this policy, ART was not universally prescribed to patients with CD4 counts above 500 cells/mm^3^. A study evaluating the intervention of Test and Treat policies demonstrated significant increases of viral suppression at the population [14]. The SFGH’s Rapid ART Program Initiative for New Diagnosis (RAPID) began in 2013 and established that any newly diagnosed or newly re-engaged patient would see an HIV health team, be offered ART (regardless of CD4 count), receive counseling, and agree on a sustainable care plan on the same day of their diagnosis/re-engagement, or within two to five days [15]. The success of the RAPID program at SFGH led to the Citywide RAPID program in 2015.

Given that San Francisco has provided many housing programs and supportive services to reduce the number of people who are unsheltered and that San Francisco was an early adopter of better clinical care programs for people living with HIV, we aimed to identify differences in causes of death (by calendar period) between people with HIV who were housed and those who were homeless to help understand the impact of these programs on fatal health outcomes. With the improvement in HIV care programs, we hypothesized that HIV-related causes of death, AIDS-defining opportunistic infections, and AIDS-defining cancers would be similar among PLWH who experienced homelessness and those who were housed. In addition, we hypothesized that deaths due to substance abuse and mental health disorders would be higher among homeless compared to housed PLWH.

## Methods

### Study sample and data collection

Included in the analyses were San Francisco residents over 12 years old, diagnosed with HIV or AIDS and reported to the San Francisco DPH, and who died from January 1, 2002 through December 31, 2016. Socio-demographic and HIV transmission characteristics, AIDS-related clinical data, and vital status were collected as part of routine HIV surveillance on all study participants. Computer matches with the National Death Index Plus provided information on multiple coded causes of death. HIV surveillance data collection is required by state law (California Health and Safety Code (HSC) 121,022 and HSC 120130), therefore Institutional Review Board (IRB) approval and consent was deemed unnecessary according to state regulations. Data used in this study is protected by state law and is not publicly available.

The International Classification of Diseases 10th edition [16] was used to code the cause of death information from death certificates. All coded causes and conditions contributing to death (including the underlying cause of death), as listed on the death certificate, were included in our multiple cause of death category. We included the most frequently occurring causes of death, both HIV/AIDS-related (presented as all HIV/AIDS-related deaths and separately as AIDS-related malignancies and AIDS-related opportunistic infections) and non-HIV-related, as our dependent variable. We also included the following causes of death regardless of the frequency of their occurrence because they are more likely to occur in people who are homeless: accident, assault, suicide, drug-related overdose, alcohol-related liver disease, and mental disorders. For AIDS-defining opportunistic infections [17], any HIV/AIDS cause of death, AIDS-defining and non-AIDS-defining cancers, heart disease (including cardiomyopathy, cerebrovascular, and ischemic disease), and mental disorders (including mental disorders due to substance abuse) composite categories were created.

Most socio-demographic characteristics in our analyses included those obtained at the time of HIV diagnosis, except for age at death, county of death, and housing status. For the purpose of HIV surveillance, the Centers for Disease Control and Prevention (CDC) uses the federal definition of homelessness. Thus, an individual is defined as homeless if their medical record denotes that the patient is not housed or homeless at the time of HIV or AIDS diagnosis, or the person’s address at diagnosis is a free postal address not connected to a residence (i.e., general delivery) or a known homeless shelter [1]. Not included in this definition are individuals with unstable (living with friends) or marginal housing (living in a single room occupancy units) [1]. For our analyses, a person was defined as homeless if periodic review of the medical record noted that the patient was homeless at the time of HIV diagnosis, AIDS diagnosis, at follow up, or at death [1].

In contingency table analyses, age at death was categorically defined by decades: 20–29, 30–39, 40–49, 50–59, 60–69, and 70–79. Race was categorized as non-Hispanic White, Hispanic, African-American, or other, including multi-race/ethnicity. HIV transmission risk category was categorized as men who have sex with men (MSM), persons who inject drugs (PWID), men who have sex with men and who also inject drugs (MSM-PWID), heterosexual, or other. Gender was classified as male, female, or transgender. Living below the federal poverty level at time of diagnosis was defined as having lived in a census tract where more than 20% of persons aged 18 years or older had a median annual household income that was below the United States poverty level [18]. Country of birth was dichotomized as USA/US Dependency or other and county of residence at death was dichotomized as San Francisco or other. Prescription of ART was dichotomized as yes or no.

### Statistical analyses

The primary independent variable was housing status dichotomized as homeless at or after HIV diagnosis or not. The primary dependent variable was multiple condition or disease-specific cause of death.

The distribution of case characteristics by housing status was compared using contingency tables, and *p* values were calculated using chi-square and Cochran-Armitage trend tests. This method was also used to explore the differences in causes of death among PLWH stratified by county of residency at time of death (San Francisco vs. non-San Francisco residents), which serves as a surrogate measure for access to San Francisco specific interventions.

To compare differences in cause-specific deaths between the housed and the homeless populations, unadjusted and adjusted Poisson regression models for binary outcomes were performed to calculate the prevalence ratios (PR’s), *p* values, and 95% confidence intervals (CI’s). The binary explanatory variable for the unadjusted model was housing status, with housed individuals being the reference group. We considered a p value of less than 0.05 significant. Adjusted models were constructed for each cause of death if the housing variable resulted in a statistically significant PR in the unadjusted model.

Adjusted regression models controlled for the following factors with their respective reference group noted in parentheses: gender (male), race (non-Hispanic white), a concurrent initial diagnosis of HIV and AIDS (yes or no), HIV transmission category (MSM), low income (yes or no), and county of residence at death (San Francisco). In addition, the regression models were adjusted for age, which was continuous per decade, and year of death, which was continuous per year. All statistical analyses were performed using SAS® software version 9.4 [19].

## Results

### Study sample characteristics

A total of 4158 deceased individuals were included in the analyses: the majority were male (87%), ages 40–59 years old at the time of death (64%), non-Hispanic White (60%), MSM (54%), had an AIDS diagnosis prior to death (87%), and San Francisco residents at the time of death (63%; Table 1). Compared to those who were housed, those who were homeless were more likely to be younger at time of death, African American, PWID, female or transgender, and living below the poverty level, and less likely to have been prescribed ART (all *p* values < 0.0001; Table 1). Those with missing baseline CD4 cell count and HIV viral load were more likely to be housed than homeless (*p* = 0.17 and *p* < 0.0001, respectively).

**Table 1:** Study characteristics among people diagnosed with HIV in San Francisco who died in 2002–2016

	Homeless *N* = 559	Housed *N* = 3599		
Characteristic	n (%)	n (%)	Chi Square *p* value	Trend Test *p* value
Year of Death				
2002–2004	148 (26.48)	845 (23.48)	0.3099	0.8104
2005–2007	115 (20.57)	869 (24.15)		
2008–2010	103 (18.43)	646 (17.95)		
2011–2013	93 (16.64)	623 (17.31)		
2014–2016	100 (17.89)	616 (17.12)		
Age at death				
20–29	20 (3.58)	39 (1.08)	**< 0.0001**	
30–39	96 (17.17)	349 (9.70)		
40–49	221 (39.53)	1084 (30.12)		
50–59	165 (29.52)	1211 (33.65)		
60–69	50 (8.94)	657 (18.26)		
70–99	7 (1.25)	259 (7.20)		
Race				
Non-Hispanic White	250 (44.72)	2227 (61.88)	**< 0.0001**	
Hispanic	76 (13.60)	441 (12.25)		
African-American	196 (35.06)	686 (19.06)		
Other	37 (6.62)	245 (6.81)		
Transmission Risk Factor				
MSM	92 (16.46)	2146 (59.63)	**< 0.0001**	
MSM-PWID	205 (36.67)	789 (21.92)		
PWID	235 (42.04)	508 (14.12)		
Heterosexual	19 (3.40)	86 (2.39)		
Other	8 (1.43)	70 (1.94)		
Gender				
Female	108 (19.32)	287 (7.97)	**< 0.0001**	
Male	411 (73.52)	3206 (89.08)		
Transgender	40 (7.16)	106 (2.95)		
Poverty at Diagnosis				
Yes	505 (90.34)	837 (23.26)	**< 0.0001**	
No	54 (9.66)	2762 (76.74)		
Country of Birth				
USA/US Dependency	502 (89.80)	3189 (88.61)	0.4050	
Other	57 (10.20)	410 (11.39)		
County of Residence at Death				
San Francisco	378 (67.62)	2253 (62.60)	**0.0220**	
Other	181 (32.38)	1346 (37.40)		
AIDS dx prior to death	483 (86.40)	3139 (87.22)	0.5929	
Prescribed ART				
Yes	424 (75.85)	3024 (84.02)	**< 0.0001**	
No	135 (24.15)	575 (15.98)		
First CD4 Count after Diagnosis (Within 6 months)				
CD4 < 200	128 (34.32)	845 (36.84)	0.1450	
CD4 200–499	139 (37.27)	905 (39.45)		
CD4 > 500	106 (28.42)	544 (23.71)		
Missing data	186	1305		
First Viral Load after Diagnosis (within 6 months)				
Viral load < 401	22 (8.33)	113 (8.96)	0.7610	
Viral load 401–3999	46 (17.42)	207 (16.42)		
Viral load 4000-49,999	102 (38.64)	454 (36.00)		
Viral load > 49,000	94 (35.61)	487 (38.62)		
Missing data	295	2338		

*P* values < 0.05 are shown in bold

### Causes of deaths by county of residency at time of death

Both San Francisco residents and non-San Francisco residents had a significant decline in deaths due to HIV/AIDS and pneumonia and a significant increase in deaths due to heart disease, ischemic disease, and non-AIDS cancer. San Francisco residents had a decrease in trend for AIDS cancer, AIDS opportunistic infections, non-cancer AIDS opportunistic infections and an increase in trend for accidental deaths, cardiomyopathy, and overdose. Non-San Francisco residents had an increase in deaths due to assault, COPD, diabetes, and mental disorders (all *p* values < 0.0001; Table 2).

**Table 2:** Temporal trends in cause-specific deaths by county of residency at time of death (San Francisco County vs. all other counties), among people diagnosed with HIV in San Francisco who died in 2002–2016

Cause of Death among San Francisco Residents			Cause of Death among Non-San Francisco Residents		
Cause of Death	N (%)	Trend Test *p* value	Cause of Death	N (%)	Trend Test *p* value
Accidental death (including drug related)		**<.0001**	Accidental death (including drug related)		0.6497
2002–2004	24 (3.64)		2002–2004	27 (8.11)	
2005–2007	61 (9.58)		2005–2007	37 (10.66)	
2008–2010	65 (13.77)		2008–2010	29 (10.47)	
2011–2013	58 (12.89)		2011–2013	27 (10.15)	
2014–2016	60 (14.56)		2014–2016	29 (9.54)	
AIDS cancer		**< 0.0001**	AIDS cancer		0.1002
2002–2004	72 (10.91)		2002–2004	33 (9.91)	
2005–2007	60 (9.42)		2005–2007	19 (5.48)	
2008–2010	33 (6.99)		2008–2010	29 (10.47)	
2011–2013	28 (6.22)		2011–2013	20 (7.52)	
2014–2016	19 (4.61)		2014–2016	15 (4.93)	
AIDS opportunistic infections (excluding AIDS cancers)		**< 0.0001**	AIDS opportunistic infections (excluding AIDS cancers)		0.0639
2002–2004	188 (28.48)		2002–2004	81 (24.32)	
2005–2007	153 (24.02)		2005–2007	79 (22.77)	
2008–2010	98 (20.76)		2008–2010	56 (20.22)	
2011–2013	79 (17.56)		2011–2013	47 (17.67)	
2014–2016	73 (17.72)		2014–2016	61 (20.07)	
Alcohol related liver disease		0.1152	Alcohol related liver disease		0.4591
2002–2004	7 (1.06)		2002–2004	5 (1.50)	
2005–2007	6 (0.94)		2005–2007	3 (0.86)	
2008–2010	6 (1.27)		2008–2010	2 (0.36)	
2011–2013	6 (1.33)		2011–2013	0 (0.00)	
2014–2016	9 (2.18)		2014–2016	4 (1.32)	
Assault		0.1028	Assault		**0.0490**
2002–2004	3 (0.45)		2002–2004	1 (0.30)	
2005–2007	5 (0.78)		2005–2007	2 (0.58)	
2008–2010	3 (0.64)		2008–2010	0 (0.00)	
2011–2013	6 (1.33)		2011–2013	2 (0.75)	
2014–2016	5 (1.21)		2014–2016	5 (1.64)	
Cardiomyopathy		**0.0080**	Cardiomyopathy		0.4488
2002–2004	13 (1.97)		2002–2004	7 (2.10)	
2005–2007	7 (1.10)		2005–2007	9 (2.59)	
2008–2010	11 (2.33)		2008–2010	7 (2.53)	
2011–2013	10 (2.22)		2011–2013	4 (1.50)	
2014–2016	18 (4.37)		2014–2016	5 (1.64)	
Cerebral vascular event		0.1230	Cerebral vascular event		0.1221
2002–2004	19 (2.88)		2002–2004	10 (3.00)	
2005–2007	16 (2.51)		2005–2007	11 (3.17)	
2008–2010	22 (4.66)		2008–2010	7 (2.53)	
2011–2013	13 (2.89)		2011–2013	6 (2.26)	
2014–2016	19 (4.61)		2014–2016	18 (5.92)	
COPD		0.1489	COPD		**0.0401**
2002–2004	37 (5.61)		2002–2004	17 (5.11)	
2005–2007	48 (7.54)		2005–2007	7 (2.02)	
2008–2010	39 (8.26)		2008–2010	12 (4.33)	
2011–2013	28 (6.22)		2011–2013	17 (6.39)	
2014–2016	36 (8.74)		2014–2016	21 (6.91)	
Diabetes		0.0804	Diabetes		**0.0003**
2002–2004	14 (2.12)		2002–2004	4 (1.20)	
2005–2007	29 (4.55)		2005–2007	11 (3.17)	
2008–2010	18 (3.81)		2008–2010	11 (3.97)	
2011–2013	25 (5.56)		2011–2013	22 (8.27)	
2014–2016	15 (3.64)		2014–2016	16 (5.26)	
HIV/AIDS		**< 0.0001**	HIV/AIDS		**< 0.0001**
2002–2004	557 (84.39)		2002–2004	259 (77.78)	
2005–2007	472 (74.10)		2005–2007	243 (70.03)	
2008–2010	314 (66.53)		2008–2010	194 (70.04)	
2011–2013	284 (63.11)		2011–2013	164 (61.65)	
2014–2016	258 (62.62)		2014–2016	183 (60.20)	
Heart disease		**0.0004**	Heart disease		**< 0.0001**
2002–2004	132 (20.00)		2002–2004	73 (21.92)	
2005–2007	128 (20.09)		2005–2007	79 (22.77)	
2008–2010	132 (27.97)		2008–2010	79 (28.52)	
2011–2013	99 (22.00)		2011–2013	89 (33.46)	
2014–2016	121 (29.97)		2014–2016	101 (33.22)	
Ischemic		**0.0408**	Ischemic		**0.0494**
2002–2004	37 (5.61)		2002–2004	25 (7.51)	
2005–2007	36 (5.65)		2005–2007	23 (6.63)	
2008–2010	36 (7.63)		2008–2010	28 (10.11)	
2011–2013	35 (7.78)		2011–2013	24 (9.02)	
2014–2016	33 (8.01)		2014–2016	34 (11.18)	
Liver disease		0.0539	Liver disease		0.2200
2002–2004	111 (16.82)		2002–2004	52 (15.62)	
2005–2007	85 (13.34)		2005–2007	41 (11.82)	
2008–2010	67 (14.19)		2008–2010	37 (13.36)	
2011–2013	56 (12.44)		2011–2013	30 (11.28)	
2014–2016	53 (12.84)		2014–2016	37 (12.17)	
Mental disorders		0.2685	Mental disorders		**0.0035**
2002–2004	61 (9.34)		2002–2004	25 (7.51)	
2005–2007	74 (11.62)		2005–2007	36 (10.37)	
2008–2010	50 (10.59)		2008–2010	28 (10.11)	
2011–2013	43 (9.56)		2011–2013	36 (13.53)	
2014–2016	31 (7.52)		2014–2016	43 (14.14)	
Mental disorders due to substance abuse		0.3142	Mental disorders due to substance abuse		**0.0061**
2002–2004	57 (8.64)		2002–2004	22 (6.61)	
2005–2007	66 (10.36)		2005–2007	33 (9.51)	
2008–2010	43 (9.11)		2008–2010	21 (7.58)	
2011–2013	42 (9.33)		2011–2013	33 (12.41)	
2014–2016	28 (6.80)		2014–2016	38 (12.50)	
Non-AIDS cancer		**< 0.0001**	Non-AIDS cancer		**0.0003**
2002–2004	79 (11.97)		2002–2004	31 (9.31)	
2005–2007	102 (16.01)		2005–2007	47 (13.54)	
2008–2010	77 (16.31)		2008–2010	43 (15.52)	
2011–2013	88 (19.56)		2011–2013	49 (18.42)	
2014–2016	83 (20.15)		2014–2016	56 (18.42)	
Overdose		**< 0.0001**	Overdose		0.3463
2002–2004	18 (2.73)		2002–2004	18 (5.41)	
2005–2007	47 (7.38)		2005–2007	19 (5.48)	
2008–2010	56 (11.86)		2008–2010	21 (7.58)	
2011–2013	51 (11.33)		2011–2013	23 (8.65)	
2014–2016	50 (12.14)		2014–2016	18 (5.92)	
Pancreatitis/ cancer		0.2176	Pancreatitis/ cancer		0.5330
2002–2004	9 (1.36)		2002–2004	2 (0.60)	
2005–2007	2 (0.31)		2005–2007	1 (0.29)	
2008–2010	2 (0.42)		2008–2010	3 (1.08)	
2011–2013	2 (0.44)		2011–2013	0 (0.00)	
2014–2016	3 (0.73)		2014–2016	1 (0.33)	
Pneumonia		**0.0045**	Pneumonia		**0.0264**
2002–2004	96 (14.55)		2002–2004	49 (14.71)	
2005–2007	81 (12.72)		2005–2007	39 (11.24)	
2008–2010	61 (12.92)		2008–2010	39 (14.08)	
2011–2013	48 (10.67)		2011–2013	19 (7.14)	
2014–2016	37 (8.98)		2014–2016	31 (10.20)	
Renal		0.4942	Renal		0.4874
2002–2004	84 (12.73)		2002–2004	25 (7.51)	
2005–2007	65 (10.20)		2005–2007	40 (11.53)	
2008–2010	48 (10.17)		2008–2010	29 (10.47)	
2011–2013	46 (10.22)		2011–2013	22 (8.27)	
2014–2016	48 (11.65)		2014–2016	33 (10.86)	
Septicemia		0.3818	Septicemia		0.6743
2002–2004	81 (12.27)		2002–2004	32 (9.61)	
2005–2007	66 (10.36)		2005–2007	34 (9.80)	
2008–2010	45 (9.53)		2008–2010	28 (10.11)	
2011–2013	47 (10.44)		2011–2013	22 (8.27)	
2014–2016	44 (10.68)		2014–2016	28 (9.21)	
Suicide		0.8411	Suicide		0.2143
2002–2004	16 (2.42)		2002–2004	9 (2.70)	
2005–2007	24 (3.77)		2005–2007	12 (3.46)	
2008–2010	22 (4.66)		2008–2010	10 (3.61)	
2011–2013	14 (3.11)		2011–2013	12 (4.51)	
2014–2016	11 (2.67)		2014–2016	13 (4.28)	
Viral hepatitis		0.0603	Viral hepatitis		0.5918
2002–2004	108 (16.36)		2002–2004	50 (15.02)	
2005–2007	97 (15.23)		2005–2007	39 (10.37)	
2008–2010	58 (12.29)		2008–2010	32 (11.55)	
2011–2013	59 (12.89)		2011–2013	31 (11.65)	
2014–2016	55 (13.35)		2014–2016	39 (12.83)	

*P* values < 0.05 are shown in bold

### Prevalence ratios – multiple causes of death

The unadjusted PR showed that homeless individuals were more likely to die from an accident, assault, mental disorder, mental disorder due to substance abuse use, overdose, and viral hepatitis compared to housed individuals. On the other hand, homeless individuals were less likely to die from diabetes, ischemic heart disease, and non-AIDS cancers.

Adjusted Poisson regression identified two cause of death categories that resulted in significantly higher adjusted prevalence ratios (aPRs) for homelessness when compared to housed individuals: mental disorders (aPR = 1.63, 95% CI 1.24, 2.14; Table 3) and the sub-category mental disorders due to substance use (aPR = 1.70, 95% CI 1.27, 2.27; Table 3). In addition, those with a history of homelessness were 37% less likely to die from non-AIDS cancers (aPR = 0.63, 95% CI 0.44, 0.89; Table 3).

**Table 3:** Unadjusted and adjusted Poisson regression prevalence ratios and 95% confidence intervals for experiencing homelessness among 4158 people diagnosed with HIV in San Francisco who died in 2002–2016

Outcome	Unadjusted PR (95% CI) for homelessness	Adjusted PR (95% CI) for homelessness
Accident (including overdose) (*n* = 417)	**1.53 (1.20, 1.95)**	1.11 (0.83, 1.49)
AIDS cancer^a^ (*n* = 328)	0.70 (0.48, 1.00)	0.89 (0.58, 1.35)
AIDS opportunistic infections (excluding AIDS cancers)^a^ (*n* = 915)	1.05 (0.87, 1.26)	
Alcoholic liver disease (*n* = 47)	1.32 (0.62, 2.83)	
Assault (*n* = 32)	**2.52 (1.17, 5.44)**	2.56 (0.90, 7.28)
Cardiomyopathy (*n* = 91)	0.89 (0.47, 1.66)	
Cerebrovascular disease (*n* = 141)	0.71 (0.41, 1.23)	
COPD (*n* = 262)	1.03 (0.72, 1.46)	
Diabetes (*n* = 165)	**0.46 (0.25, 0.85)**	0.60 (0.30, 1.17)
Heart disease^a^ (*n* = 1033)	0.86 (0.71, 1.04)	
HIV/AIDS^a^ (*n* = 2928)	0.95 (0.85, 1.06)	
Ischemic heart disease (*n* = 311)	**0.59 (0.39, 0.88)**	0.87 (0.55, 1.38)
Liver disease (*n* = 569)	1.21 (0.97, 1.52)	
Mental disorders^a^ (*n* = 427)	**2.15 (1.73, 2.78)**	**1.63 (1.24, 2.14)**
Mental disorders due to substance use (*n* = 383)	**2.24 (1.79, 2.82)**	**1.70 (1.27, 2.27)**
Non-AIDS cancer^a^ (*n* = 655)	**0.43 (0.31, 0.59)**	**0.63 (0.44, 0.89)**
Overdose (*n* = 321)	**1.57 (1.19, 2.07)**	1.06 (0.76, 1.47)
Pancreatic disease (*n* = 25)	1.23 (0.42, 3.57)	
Pneumonia (*n* = 500)	1.12 (0.87, 1.43)	
Renal disease (*n* = 440)	1.12 (0.86, 1.45)	
Septicemia (*n* = 427)	1.14 (0.87, 1.48)	
Suicide (*n* = 143)	0.70 (0.40, 1.21)	
Viral Hepatitis (*n* = 564)	**1.32 (1.06, 1.65)**	0.88 (0.68, 1.38)

Poisson regression adjusted for gender, race, age, concurrent HIV and AIDS diagnosis, HIV transmission risk, income status at diagnosis, year of death, prescription of ART, and San Francisco resident at time of death

^a^Composite causes of death

*P* values < 0.05 are shown in bold

## Discussion

We observed that PLWH who experienced homelessness were more likely to have mental disorders, and in particular mental disorders related to substance use, as a contributory cause of death, as hypothesized. We also saw a lower risk of death from non-AIDS cancer among homeless PLWH compared to those who were housed. There was no association with other causes of death between the housed and homeless population, including HIV/AIDS, non-cancer AIDS opportunistic infections, and AIDS cancer, suggesting that the benefit of access to early and sustained ART among PLWH was independent of housing status.

Both substance use and mental disorders are common among persons living with HIV and we observed deaths due to drug overdoses increased over time in San Francisco. Studies reported that approximately 40% of PLWH have some type of mental disorder, and 21–37% of PLWH experience substance use disorders [20, 21]. Substance use and mental disorders have been associated with decreased ART adherence and worse health outcomes [22–24]. PLWH with a history of injection drug use were reported to be less likely to access ART in several studies [25, 26]. Another study in Philadelphia observed that PLWH who had mental illness were less likely to achieve viral suppression than PLWH without mental illness (adjusted OR 0.65, 95% CI 0.47, 0.91) [27]. These findings indicate that more outreach and/or services are needed to help those with mental health and substance use disorders.

Understanding how homelessness affects the health of PLWH who have co-occurring behavioral conditions is critical [28]. A study in New York City assessed whether the provision of supportive housing improves AIDS-free survival among chronically homeless PLWH with substance use or mental health disorders. The investigators observed a greater risk of death or AIDS diagnosis in unhoused compared to housed PLWH (adjusted hazard ratio 1.84, 95% CI 1.40, 2.44) [29]. Because housing is a key social determinant of health for PLWH [7, 30, 31] a continued investment in supportive housing is necessary to meet the U.S. End the HIV Epidemic goals [32].

Even in the absence of HIV, homeless individuals are more likely to need psychiatric services. Compared to housed patients, homeless patients with access to San Diego County mental health services were four times more likely to use hospitalization and emergency services to treat serious psychiatric issues [33]. This study also found that patients with schizophrenia and bipolar disorder were 1.6 and 2.4 times, respectively, more likely to be homeless when comparing to those with major depression [33].

The combination of homelessness, HIV, and mental illness is an area of synergy that requires more attention by health care providers and policy makers. There have been efforts to stabilize the marginally housed and improve mental health services in San Francisco through DAH, Care Not Cash, and MHSA. Additionally, in 2018, California voters passed Prop 2, which allows revenue generated from MHSA to be used specifically for housing homeless people in need of mental health services and San Francisco voters passed Prop C, which will tax businesses to generate funds for housing and homeless services. However, additional public programs may be needed to improve mental health services and substance abuse resources for homeless PLWH in particular.

Many studies have reported an increased incidence of non-AIDS cancer among PLWH due to increasing life expectancy and higher rates of co-infection with oncogenic viruses like Epstein Barr Virus, Human Papillomavirus, and Hepatitis B [34–39]. However, PLWH who were homeless in our study were less likely to die from non-AIDS cancers (such as lung, liver, anal, colon, pancreatic, rectal, leukemia, and Hodgkin lymphoma) most likely because of other competing causes of death, such as mental disorders and substance use. Another potential explanation is that people who are homeless are less likely to get diagnosed with cancer due to limited access to health care in general and cancer screening in particular.

There are several limitations to this study. First, the cause of death data, as noted on the death certificates, may be inaccurate or incomplete, an issue which has been shown to commonly occur in the United States [40–42]. Inaccurate reporting would lead to misclassification bias and incomplete reporting would lead to imprecision. Second, information on housing status prior to HIV diagnosis was not recorded in the San Francisco HIV/AIDS registry; therefore, individuals who were homeless prior to being diagnosed with HIV were categorized as housed. Nevertheless, being homeless prior to HIV diagnosis likely had less of an influence on causes of death compared to any recent history of homelessness since diagnosis. Additionally, individuals who were homeless at the time of diagnosis and were subsequently housed were categorized as homeless, which would bias our results towards the null. Third, we did not have individual-level information on use of HIV and housing-related programs. Instead, we used calendar time and county of residence at death as a surrogate to measure access to San Francisco and California specific HIV and housing interventions. We also did not have individual-level data on ART adherence or discontinuation, but rather assumed that all deaths occurred in the era of wide ART availability. Last, the definition of homeless used by HIV surveillance excludes those who are marginally housed and thus combining these individuals with those who have stable housing might have diminished the impact of housing status on the various causes of death.

Despite these limitations, our study has several strengths. First, the use of cause of death data from death certificates was standardized and used in other studies and thus our results can be more easily compared. Second, the systematic collection of housing status both at the time of diagnosis and through subsequent medical chart reviews provide information that is unique and not widely available elsewhere. Third, the San Francisco HIV/AIDS surveillance registry was 95–99% complete^1^ and thus is less subject to reporting bias. Last, the study was relatively large, population-based, and spanned a 15-year time period, all in the era of effective ART making our observations generalizable, comprehensive, and timely.

## Conclusions

In summary, our analyses identified the life-threatening conditions and diseases that homeless people living with HIV may experience and these results can be used to direct policy decisions aimed at reducing mortality among this population. Such policies need to involve multiple approaches such as increased mental health services and housing in the homeless PLWH population. However, more research is needed to quantitatively evaluate use of specific mental health services provided in San Francisco, especially in light of the upcoming housing support for persons experiencing homelessness from the recently enacted State and local ballot propositions. Treatments for HIV have achieved remarkable success in preventing HIV-related deaths; as such the social determinants of health that still impact mortality must continue to be monitored and evaluated so that appropriate and effective policies and interventions can be implemented to reduce mortality among all persons living with HIV.

## Data Availability

The datasets generated and/or analyzed during the current study are not publicly available as these HIV surveillance data are protected under California state law HSC 121022 and HSC 120130.

## Abbreviations

AIDS: Acquired immunodeficiency syndrome
aPR: Adjusted prevalence ratio
ART: Antiretroviral therapy
CD4: CD4 T lymphocyte
CDC: Centers for Disease Control and Prevention
CI: Confidence interval
DAH: Direct access to housing
DPH: Department of Public Health
HIV: Human immunodeficiency virus
HSC: Health and safety code
IRB: Institutional Review Board
MHSA: Mental Health Services Act
MSM: Men who have sex with men
MSM-PWID: Men who have sex with men and who also inject drug
OR: Odds ratio
PLWH: People living with HIV
PR: Prevalence ratio
PWID: Person who injects drugs
RAPID: Rapid ART program initiative for new diagnosis
SFGH: San Francisco General Hospital
